# Supplementary material for: Achieving High-Strength and Toughness in a Mg-Gd-Y Alloy Using Multidirectional Impact Forging
Source: Materials (Basel). 2022 Feb 17;15(4):1508. doi: 10.3390/ma15041508 (PMC8876578; doi:10.3390/ma15041508)
Supplement: Supplementary file 1 [file materials-15-01508-s001.zip › materials-1570799-supplementary.pdf]

materials

MDPI

Article

Achieving High-Strength and Toughness in a Mg-Gd-Y Alloy Using Multidirectional Impact Forging

Songhe Lu [1,2,†], Di Wu [2,†], Ming Yan [1] and Rongshi Chen [2,*]

[1] Academy for Advanced Interdisciplinary Studies, Southern University of Science and Technology, Shenzhen 518055, China; lush3@sustech.edu.cn (S.L.); yanm@sustech.edu.cn (M.Y.)
[2] Shi-changxu Innovation Center for Advanced Materials, Institute of Metal Research, Chinese Academy of Sciences, 72 Wenhua Road, Shenyang 110016, China; dwu@imr.ac.cn
* Correspondence: rschen@imr.ac.cn; Tel.: +86-24-23926646; Fax: +86-24-23894149
† These authors contributed equally to this work.

Elektron Wrought Alloys

Typical chemical composition – major alloying elements %	Elektron alloy	Tensile properties[B]			Compressive properties		Fatigue properties[D]		Hardness	Description
		0.2% proof stress (MPa)	Tensile strength (MPa)	Elongation[C] (%)	0.2% proof stress (MPa)	Compressive strength (MPa)	Unnotched (MPa)	Notched (MPa)	v.p.n.	
Y 5.25<br>Nd 3.5[A]<br>Zr 0.5	WE54 Extruded bars and sections									High strength at elevated temperatures particularly in the fully heat treated condition.
	Precipitation treated	(180)	(280)	(6)	–	–	–	–	75–95	
	Fully heat treated	(160)	(250)	(6)	–	–	–	–	75–95	
	Forgings[E]									
	Precipitation treated	(165)	(310)	(4)	–	–	–	–	–	
	Fully heat treated	(170)	(260)	(6)	–	–	–	–	–	
Y 4.0<br>Nd 3.0[A]<br>Zr 0.5	WE43 Extruded bars									High strength aerospace alloy at elevated temperatures particularly in the fully heat treated condition.
	Precipitation treated	(160)	(245)	(6)	–	–	–	–	75–95	
	Fully heat treated	(130)	(230)	(7)	–	–	–	–	75–95	
	Forgings[E]									
	Precipitation treated	(155)	(285)	(6)	–	–	–	–	–	
	Fully heat treated	(165)	(265)	(6)	–	–	–	–	–	

Figure S1. Room temperature tensile properties of extruded bars and forged billets of commercial WE43 and WE54 alloys produced by Elektron Ltd (London, UK).

Magnesium Elektron Elektron® 43 Magnesium Wrought Alloy

Categories: Metal; Nonferrous Metal; Magnesium Alloy

Material Notes: High strength wrought Mg alloy available as rolled plate, forging feedstock, and extrusions. It is a wrought evolution of the casting alloy Elektron WE43. Elektron 43 uses zirconium grain refining and has successfully undergone flammability tests by the FAA for use in aircraft seat frames. It develops maximum strength in the T5 condition.

Vendors: Magnesium Elektron is the world leader in high strength magnesium alloys in rolled, extruded and cast forms. Phone (303) 589-3189 or visit www.magnesium-elektron.com.

Click here to view all available suppliers for this material.

Please click here if you are a supplier and would like information on how to add your listing to this material.

Physical Properties	Metric	English	Comments
Density	1.84 g/cc	0.0665 lb/in³	

Mechanical Properties	Metric	English	Comments
Hardness, Brinell	70 - 90	70 - 90	
Tensile Strength	296 MPa	43000 psi	Extruded Bar - Transverse
	331 MPa	48000 psi	Extruded Bar - Longitudinal
	345 MPa @Thickness 1.02 - 38.1 mm	50000 psi @Thickness 0.0400 - 1.50 in	Rolled Plate
	345 MPa @Thickness 38.13 - 76.2 mm	50000 psi @Thickness 1.501 - 3.00 in	Rolled Plate
Tensile Strength, Yield	179 MPa @Strain 0.200 %	26000 psi @Strain 0.200 %	Extruded Bar - Transverse
	214 MPa @Strain 0.200 %	31000 psi @Strain 0.200 %	Extruded Bar - Longitudinal
	255 MPa @Strain 0.200 %, Thickness 38.13 - 76.2 mm	37000 psi @Strain 0.200 %, Thickness 1.501 - 3.00 in	Rolled Plate
	276 MPa @Strain 0.200 %, Thickness 1.02 - 38.1 mm	40000 psi @Strain 0.200 %, Thickness 0.0400 - 1.50 in	Rolled Plate
Elongation at Break	11 %	11 %	Extruded Bar - Transverse
	14 %	14 %	Extruded Bar - Longitudinal
	12 % @Thickness 1.02 - 38.1 mm	12 % @Thickness 0.0400 - 1.50 in	Rolled Plate
	14 % @Thickness 38.13 - 76.2 mm	14 % @Thickness 1.501 - 3.00 in	Rolled Plate

Figure S2. Room temperature tensile properties of rolled plate and extruded bar of commercial WE43 alloys produced by Elektron Ltd (London, UK).
